# New Trends in the Impact of Periodontal Treatment on Early Cardiovascular Diseases Outcomes: Insights and Future Perspectives

**DOI:** 10.31083/j.rcm2410287

**Published:** 2023-10-08

**Authors:** Mariacristina Amato, Saturnino Marco Lupi, Alessandro Polizzi, Simona Santonocito, Gaia Viglianisi, Marco Cicciù, Gaetano Isola

**Affiliations:** ^1^Department of General Surgery and Surgical-Medical Specialties, School of Dentistry, University of Catania, 95124 Catania, Italy; ^2^Department of Clinico-Surgical, Diagnostic and Pediatric Sciences, School of Dentistry, University of Pavia, 27100 Pavia, Italy

**Keywords:** periodontitis, periodontics, periodontal treatment, atherosclerosis, cardiovascular disease, endothelial dysfunction

## Abstract

Cardiovascular diseases represent the primary worldwide cause of mortality, and periodontitis is the main cause of tooth loss. The incidence of atherosclerotic disease has been reported to be higher in individuals affected by periodontitis than in individuals without, regardless of many common risk factors are present. Various pathogenetic models have been presented to clarify the close correlation between these two diseases. First, periodontal bacteria and their toxins can enter the circulation both during dental procedures and normal activities such as eating and teeth brushing. Periodontal bacteria may indirectly contribute to coronary artery disease (e.g., by causing immunological reactions) or directly by damaging coronary arteries. Periodontal treatment significantly reduces periodontal pathogens such as *Porphyromonas gingivalis* (*Pg*) or *Actinobacillus actinomycetemcomitans* (*Aa*) in deep periodontal pockets. Moreover, periodontal treatment may lower blood inflammatory mediators, enhance the lipid profile, and cause favourable changes in various surrogate markers for cardiovascular disease. The way in which oral bacteremia and periodontal inflammation cause atherosclerosis is still unclear and needs further studies. The real effectiveness of periodontal treatment in preventing cardiovascular events is a topic of current interest. In this regard, this review article explores new insights and provides an indication of future directions on the function of periodontal inflammation and oral bacteria in the incidence and progression of atherosclerosis and cardiovascular diseases, with the main focus on assessing the impact of periodontal treatment on cardiovascular disease outcome biomarkers.

## 1. Introduction

During the 20th century, there was an increase in death and disability caused by noncommunicable diseases (NCDs) [1, 2, 3]. Among such NCDs, cardiovascular disease (CVD) now represents the main cause of morbidity and death worldwide [4]. CVD fatalities are due to different states, such as hypertensive heart disease (which leads to heart failure) [5, 6, 7], stroke [5, 6, 7, 8], ischemic heart disease (IHD) [5, 6, 7, 8], atrial fibrillation and cardiomyopathy [3, 5, 6, 7, 8], and rheumatic heart disease (RHD) [5, 6, 7, 8]. The increasing number of deaths caused by CVD is attributed to two factors: population growth and the ageing of the population. It is a challenge to decrease CVD mortality, in particular, in 2013 the WHO launched the ambitious project of 25 × 25 Global Action Plan, whose objective is to decrease NCD- related premature mortality by 25% by 2025, including CVD [4]. To obtain such a score, interfering with the recognized epidemiologic factors of such diseases is important. In fact, it has been demonstrated that periodontal inflammation and oral microorganisms influence the incidence and progression of atherosclerosis and cardiovascular diseases. Actually, the correlation between oral health and systemic health is not new. It is notorious that patients with an elevated risk of endocarditis have to follow antimicrobial prophylaxis when they undergo dental procedures. That is done to avoid endocarditis following such dental therapy in order to allow the passage of oral microorganisms into the circulatory system [9, 10].

Periodontitis is a disease characterized by chronic inflammation that affects the tooth-supporting structures such as the periodontal ligament, cementum and alveolar bone. It is a global disease; in fact, according to WHO, it affects 10–15% world’s population. It is a multifactorial disease involving biofilm, genetic predisposition and other additional factors, such as smoking and systemic diseases. Among the systemic diseases that contribute to the development of periodontitis, there is cardiovascular disease. Thus, it means that periodontitis and cardiovascular disease are linked, influencing each other reciprocally. Nevertheless, the underlying mechanisms that explain the correlation between the two conditions are not totally clear, and the scientific community is investigating them [11]. Therefore, the aim of this review is to describe the current knowledge regarding the influence of periodontal health on cardiovascular health, identifying how periodontal treatment can enhance cardiovascular health.

## 2. Atherosclerosis and Related CVDs

**Fig. 1.**
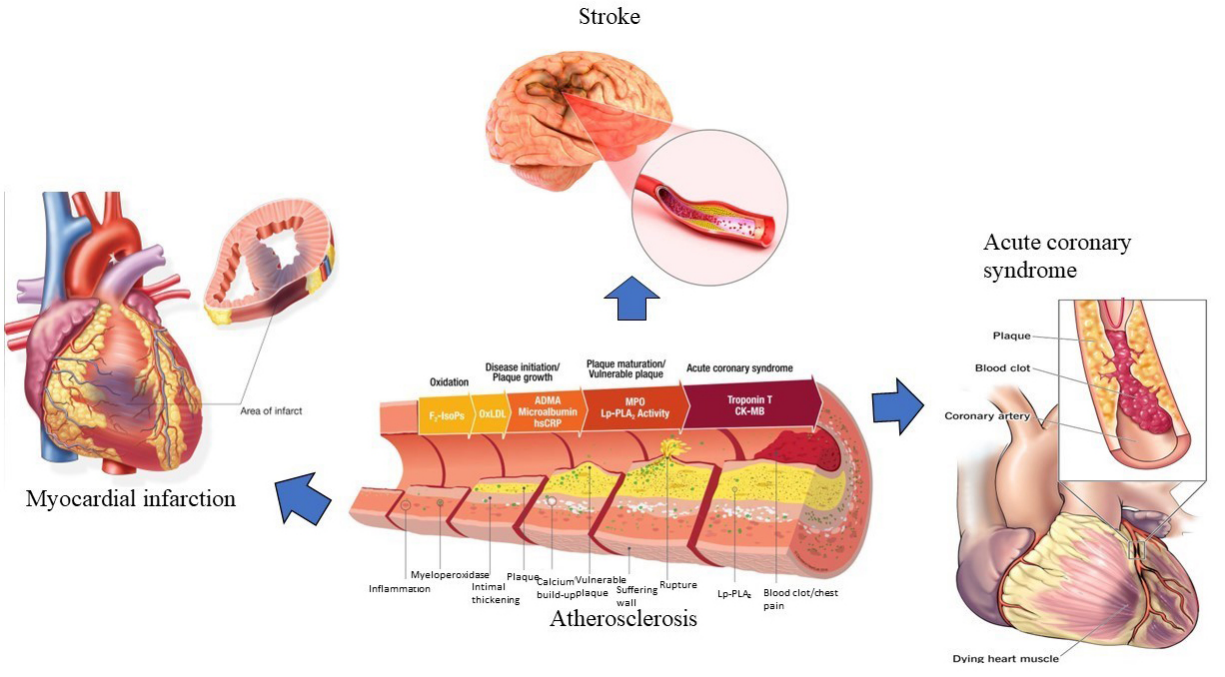
**Atherosclerosis and the related CVDs.** Atherosclerosis development and progression may lead to other CVDs, including myocardial infarction, stroke and acute coronary syndrome. CVDs, cardiovascular diseases; IsoPs, isoprostanes; ADMA. asymmetric dimethylarginine; hsCRP, high sensitivity C-reactive protein; Lp-PLA2, lipoprotein-associated phospholipase A2; OxLDL, oxidized low-density lipoprotein; CK-MB, creatine kinase-MB.

Atherosclerosis is a chronic disease affecting arteries of large and medium size. It is considered the pathogenic basis of most CVDs, including stroke, acute coronary syndromes, and myocardial infarction [12] (Fig. 1). It manifests as the deposit of fatty materials and the constitution of atherosclerotic plaques in the arteries. Its pathophysiology is based on the activities and the transformation of groups of cells, in particular endothelial cells, vascular smooth muscle cells, macrophages, T cells and dendritic cells. First, the endothelium is stimulated; then, as the disease proceeds, some areas of the endothelium are damaged and exposed, with platelets reaching them and accumulating there. After that, monocytes move from the blood to the subintima of the artery, they enter the cells, alter lipoproteins and differentiate into foam cells. Vascular smooth muscle cells change their phenotype from contractile to synthetic, so they replicate, migrate and generate a substantial extracellular matrix that composes the fibrous cap of atherosclerotic plaques. Such cells are also able to assume lipids and differentiate into foam cells. In addition, the immune response driven by T cells and dendritic cells (DCs) also plays a central role in the pathological advance of atherosclerosis [13, 14, 15, 16].

Atherosclerosis is a multifactorial disease, and the causing factors are hypertension, hyperlipidemia, smoking, diabetes mellitus, chronic inflammation, family history, immunosuppression, socioeconomic status, age, stress, poor nutrition, physical inactivity and obesity [17]. Besides these well-known risk factors, there are other issues that impact the development and the progression of atherosclerosis. Even though most recent studies have only investigated the impact of intervention on the aforementioned risk factors, atherosclerosis is still a public health problem [18], suggesting that other factors may be included and should be considered, including the co-presence of periodontitis, which we will analyse in this review.

Atherosclerosis is linked with the following CVDs: acute myocardial infarction, which is myocardial necrosis due to prolonged ischaemia [19] and stroke, which indicates an acute event of focal dysfunction of the brain, retina, or spinal cord lasting more than 24 hours or of any duration assessed by computed tomography/magnetic resonance imaging or autopsy with focal infarction or haemorrhage relevant to the symptoms [20]. Moreover, atherosclerosis shares a lot of risk factors with cardiac calcification [21, 22]. Chronic inflammation is a significant factor in developing and progressing various diseases, including atherosclerosis and cardiac calcification. Both atherosclerosis and cardiac calcification have been linked to chronic kidney disease (CKD) and are often more severe in patients undergoing dialysis. CKD is associated with a state of chronic inflammation, which can accelerate the development of atherosclerosis and contribute to the severity of coronary calcification. Additionally, CKD itself is a risk factor for cardiovascular disease, and the presence of cardiac calcification further increases the risk of adverse cardiovascular outcomes in these patients [23, 24, 25, 26, 27].

## 3. Periodontitis

**Fig. 2.**
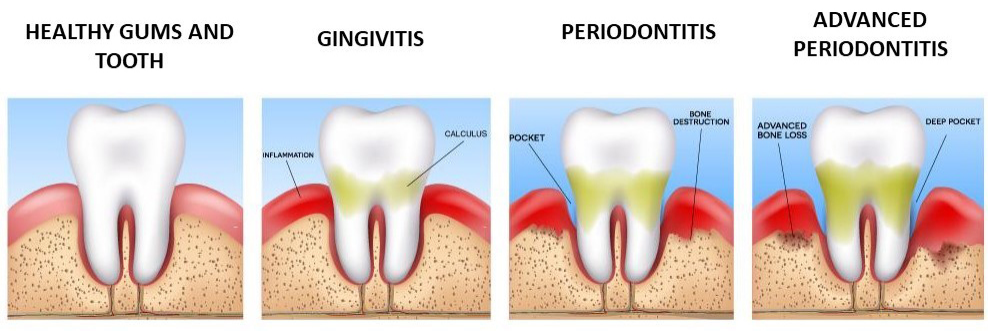
**The progression of periodontal disease. **Gingivitis affects gums only. Periodontitis affects gums and alveolar bone, giving rise to pockets and bone resorption and in advanced periodontitis there are deeper pockets and advanced bone resorption that leads to tooth loss.

Periodontitis is a chronic inflammatory disorder that affects the tissues surrounding and supporting the tooth, including cementum, periodontal ligament and alveolar bone [28, 29, 30] (Fig. 1). It is a multifactorial disease whose main causing factors are represented by the dental biofilm and the genetic predisposition. In fact, the presence of biofilm is necessary but not sufficient to start the development of periodontal disease, in fact, subsequent to infection, in genetically predisposed people, an aberrant inflammatory response is generated, causing damage of the associated tissues. The presence of the biofilm induces such a response, which manifests clinically by augmented values of probing depth (PD), loss of clinical attachment level (CAL), gingival bleeding and tooth mobility, and radiographically by alveolar bone resorption [29, 30, 31] (Fig. 2). The plaque is directed apically. Bone resorption follows such a direction. Thus, coronal bone loss is always more severe than apical bone loss [32]. Other etiological factors that may induce periodontitis are the co-presence of other systemic diseases, like diabetes mellitus, cardiovascular disease, and rheumatoid arthritis [29]; behavioural factors, like smoking, lack of physical activity, stress, obesity and diet; the influence of the diet is emerging from the latest studies, even if there isn’t still strong evidence [33]. It is worth highlighting that all the systemic diseases that influence periodontitis share an inflammatory profile, suggesting that a state of inflammation in an organ may influence other anatomical districts.

## 4. Cardiovascular Diseases and Periodontitis

The risk of developing atherosclerotic cardiovascular disease (ACVD) in periodontal patients was assessed in 2012 during the workshop between the European Federation of Periodontology (EFP) and the American Academy of Periodontology, in which the interaction between periodontitis and systemic diseases was analyzed and discussed [34]. It is worth mentioning that, at first, the link between periodontal disease and ACVD emerged from observational studies, in fact, it was detected that acute coronary syndromes affected patients that were assisted in the emergency rooms had worse oral hygiene than healthy controls [35]. Since then, the studies to demonstrate the evidence of such a correlation has increased.

Epidemiological data highlight the evidence of the augmented risk of ACVD in patients with PD compared to healthy periodontal patients [36]; for this reason, the hypothesized biological correlation between periodontitis and CVD should be discussed, in particular, ACVD. As mentioned before, the main shared factor between ACVD and cardiac calcification is chronic inflammation which is also related to periodontitis since it is considered a source of chronic inflammation that leads to adverse cardiovascular effects and consequences [37]. In fact, for example, the study conducted by Pressman *et al*. [37] assessed the correlation between cardiac calcification and periodontal disease. They conducted a cross-sectional study that involved subjects from two different sites, one in the USA and the other in Japan. The study aimed to investigate the relationship between periodontal disease severity, as assessed by detailed dental examinations, and echocardiographic calcification. To assess the severity of periodontal disease, the researchers employed semiquantitative scoring systems. These scoring systems likely involved evaluating various parameters related to periodontal health, such as probing depth, attachment loss, bleeding on probing, and presence of dental plaque or calculus. By using a semiquantitative approach, the researchers assigned scores to each parameter, allowing for a standardized assessment of periodontal disease severity. Simultaneously, the study participants also underwent clinical echocardiograms, which are ultrasound examinations of the heart. Echocardiographic calcification refers to the presence and severity of calcified deposits within the heart structures as detected by the echocardiogram. This can include calcification of the heart valves, the coronary arteries, or other cardiac structures. By comparing the periodontal disease severity scores with the echocardiographic calcification findings, the researchers aimed to determine if there was any correlation or association between the two. The results showed that the two pathological conditions were linked, by identifying a significant correlation between the degree of periodontal disease, and cardiac calcification. Furthermore, greater periodontal records were associated with greater degrees of calcification. Considering the results of this study which report a correlation between chronic inflammation,cardiac conditions and periodontitis, it is clear that periodontitis is linked with CVDs. Moreover, in 2012, scientists, supported by *in vitro*, pre-clinical and clinical study evidence, concluded that CVD is caused directly or indirectly by translocated oral microbiota of periodontal patients. Such microbiota create a systemic inflammatory environment, which in the end, enhances the advancement of atherothrombogenesis [34]. However, data for this is limited, so further studies are needed to identify and analyze the involved molecules andbacteria and their role in these pathophysiological mechanisms.

### 4.1 Periodontal Bacteria in the Etiopathogenesis of Periodontitis

**Fig. 3.**
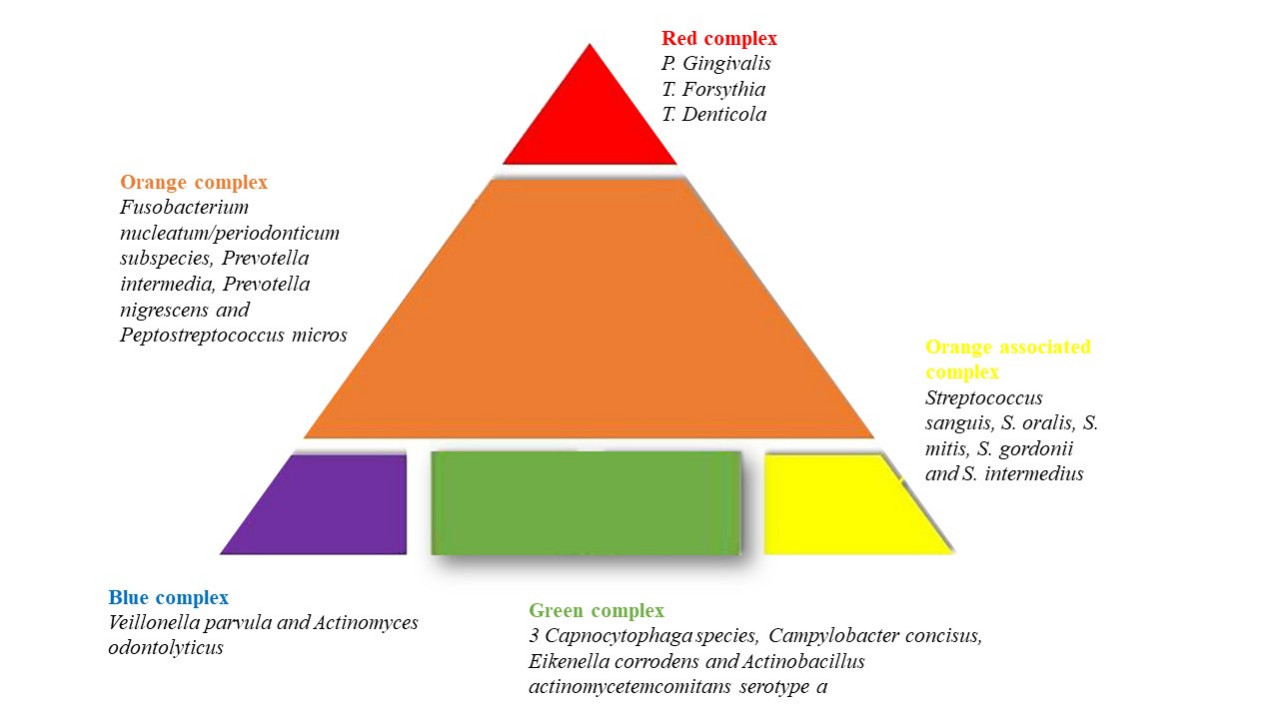
**Complexed in which are organized the bacteria in subgingival plaque. **Each complex is associated a color. Bacteria are divided in such complexes according to their characteristics.

Bacteria are organized in complexes in subgingival plaque (Fig. 3). Complex 1 is composed of *Tannerella Phorsithia*, *Porphyromonas gingivalis* and *Treponema denticola * [38]; complex 2 includes members of the *Fusobacterium nucleatum/periodonticum* subspecies, *Prevotella intermedia*, *Prevotella nigrescens* and *Peptostreptococcus micros*; complex 3 consists of *Streptococcus sanguis*, *S. oralis*, *S. mitis*, *S. gordonii* and *S. intermedius*; complex 4 contains 3 *Capnocytophaga species*, *Campylobacter concisus*, *Eikenella corrodens* and *Actinobacillus actinomycetemcomitans serotype a*. and complex 5 consists of *Veillonella parvula* and *Actinomyces odontolyticus*. Among them, complex 1 is related to periodontal disease [38]. Periodontitis, as previously mentioned, is a multifactorial disease, nevertheless, the causing bacteria, organized in biofilm, represent an important etiological factor, on which the therapy is based. Such bacteria of the 1st complex are present in big quantities and more frequently in periodontal patients than healthy ones [39, 40].

*Porphyromonas gingivalis* is part of the phylum Bacteroidota and is a nonmotile, Gram-negative, rod-shaped, anaerobic, pathogenic bacterium - it is the most recognized periodontal pathogen [41]. Even if the gingival epithelium represents the first barrier, whose role is to impede penetration of pathogens, *P. gingivalis* is able to penetrate it, to reach the underlying connective tissue [42, 43] by destroying transmembrane proteins of the epithelium [44, 45, 46]. In this way, *P. gingivalis* invades and destroys periodontal tissues, causing an inflammatory response that manifests through an increase of periodontal parameters (PD, CAL) and alveolar bone resorption. Moreover, *P. gingivalis* has great survival skills, in fact it manages to evade the immune system of the host. Intracellular *P. gingivalis* can utilize autophagy for its survival in gingival epithelial cells (GECs) [47, 48, 49, 50], and also it can manipulate differentiation and immune responses of T cells that provide a guarantee for its survival in the host [51]. In particular, SerB, a phosphoserine phosphatase, impedes interleukin-8 (IL-8) action and may disorganize other epithelial-cell homeostasis programs and Specific lipid A structures *Porphyromonas gingivalis* lipopolysaccharide (LPS) blocks the reply of Toll-like receptor 4 (TLR4) to other bacteria [52, 53, 54].

*Treponema denticola* is anaerobic pathogenic bacteria and belongs to spirochetes. Its by-products work on mucosal cells and immune system cells, initiating cell impairment and liberation of cellular damaging factors to the periodontal tissues [42]. It adheres to epithelial cells and fibroblasts and also to other periodontal pathogens, especially *P. gingivalis*, such interaction plays an important role in starting and enhancing periodontitis.

### 4.2 Periopathogens in the Etiopathogenesis of Cardiovascular Diseases

**Fig. 4.**
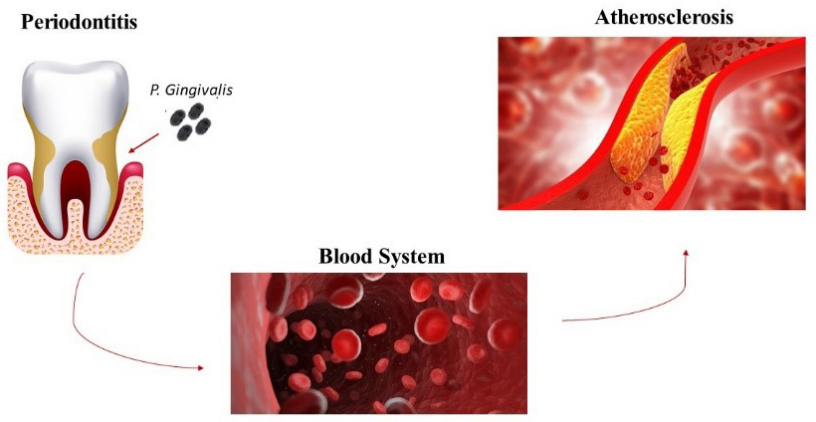
***P. gingivalis* and its behavior.** P. gingivalis is the main oral pathogen implicated in the pathogenesis of atherosclerosis. After daily oral activities, such as brushing or professional treatment of the oral cavity it manages to go into the blood system which is important to develop atherosclerosis.

As previously discussed, there is a strong interaction between Periodontal disease and CVDs. Periodontal bacteria manage to go into the circulatory system via epithelium ulcers and lymphatic vessels after daily activities, such as brushing and chewing or after professional intervention, such as subgingival scaling and surgical periodontal therapy; then they colonize other organs, leading to disturbances or diseases far from the oral cavity, in which they have already caused periodontitis [55] (Fig. 4). Epidemiological and clinical studies have revealed that periodontal disease is associated with carotid atherosclerosis [56, 57]. Periodontitis-affected patients have a higher risk of AS/CVD, and its risk ratio ranges from 1.074 to 1.213, 95% confidence interval (CI) [58, 59, 60, 61].

A clinical study [62] investigated the composition of vascular biopsies taken from patients with vascular diseases (VD; i.e., abdominal aortic aneurysms, atherosclerotic carotid, and common femoral arteries), with or without chronic periodontitis (CP), focusing in the analysis of bacteria and their DNA. The obtained bacterial DNA was amplified by polymerase chain reaction (PCR), and the amplicons were duplicated into Escherichia coli, sequenced, and organized by class. Ten vascular biopsies were randomly chosen from the CP group and underwent scanning electron microscopy (SEM) to identify and to observe the bacteria. Moreover, of the subgingival plaque samples from CP patients, 10 were picked to undergo Checkerboard DNA-DNA hybridization in order to verify the existence of red complex bacteria in such samples. The results demonstrated that high levels of bacteria were present in the cardiovascular biopsies of CP group, those bacteria were from the oral cavity and also from the gut, confirming that periopathogens have an impact in the advance of CDV, but in association to them, there are other bacteria too, coming from the gut.

Periodontal bacteria have been found in human atherosclerosis plaque lesions, including *P. gingivalis*, *Aggregatibacter actinomycetemcomitans* and *Tannerella forsythia * [63, 64, 65]. Nevertheless, in the last years, the research has focused on the correlation of the main represented periopathogen, *P. gingivalis* and the development and progression of atherosclerosis. Several studies have explored the function of *P. gingivalis* in the pathogenesis of atherosclerosis [66, 67, 68, 69, 70, 71, 72] (Fig. 4).

#### 4.2.1 Porphyromonas Gingivalis

One of the proposed mechanisms of *P. gingivalis* is to activate endothelial oxidative stress and promote the inflammation response; this leads to endothelial dysfunction which is the precursor to atherosclerotic lesions [73]. *P. gingivalis* manages to do that through the TLRs-nuclear factor kappa-light-chain-enhancer of activated B cells (NF-kB) axis. In fact, *P. gingivalis* LPS has been recognized thanks to TLRs, which enhance the downstream signaling pathway NF-kB and the subunit p65, favouring oxidative stress [74]. *P. gingivalis* promotes the inflammatory response by increasing the levels of the following inflammatory factors IL-1β, IL-6, tumor Necrosis Factor alpha (TNFα), and interferon-gamma (IFN-γ) in the endothelial cells [75, 76]. It also causes the increase of endothelial cell receptors that favours the recruitment and the subsequent adhesion of monocytes, initiating, in this way, the inflammatory response [16].

Moreover, *P. gingivalis* causes endothelial cell permeability by upregulating IL-8, which is directly involved in the increase of such permeability through the activation of the NF-kB pathway. Its gingipains promote endothelial destruction by causing the disorganization of adhesion molecules like vascular-endothelial cadherin (VE)-cadherin and N-cadherin and favour the internalization of VE-cadherin in endothelial cells, which is also involved in the regulation of endothelial cells permeability [77, 78]. The increase of endothelial permeability enhanced by *P. gingivalis*, explains why it is not the only type of bacteria found in atherosclerotic lesions, in fact, by augmenting the penetrability of the endothelium, other circulating bacteria may reach such lesions. Another interesting fact of the impact of *P. gingivalis* in the pathogenesis of atherosclerosis is that it is able to induce endothelial cells apoptosis, leading to a damaged endothelium. Under such damaged endothelium, circulating leukocytes and low-density lipoprotein (LDL) accumulate, promoting the development of atherosclerosis. The biochemical mechanism by which *P. gingivalis* manages to induce cell apoptosis is related to the induction of an increase in pro-apoptotic factors, including proteins Bcl-2-associated X protein and caspase 3 (CAS3) and the decrease of anti-apoptotic factor, protein Bcl-2. In addition, *P. gingivalis* causes endothelial cell apoptosis by activating the caspase-8 death receptor and the caspase-9 mitochondrial-dependent apoptosis pathway and by promoting the DNA fragmentation activated by caspase-3; it enhances the expression of many growth arrest- and DNA damage-associated genes including inducible gene 153, glucose-regulated protein 78 and caspase-12 [46, 74, 79, 80, 81]. *P. gingivalis* reaches the endothelium and survives due to autophagy; in this way, it can preserve its virulence, but the underlining mechanism needs to be clarified [82]. It is interesting to mention the interface between *P. gingivalis* and vascular smooth muscle cells (VSMCs). Such cells are normally quiet under good health conditions of the cardiovascular system, but when the blood vessels suffer damage, they activate from a contractile phenotype into a synthetic phenotype. This means that they begin migrate, proliferate and carry out proteosynthesis, which results in stenosis or obliteration of the vascular lumen [83]. The change into this synthetic phenotype can also be related to the action of *P. gingivalis*; in fact it can enhance VSMC proliferation and migration from the middle layer to the inner layer of the blood vessel, giving rise to the progression of Atherosclerosis. *P. gingivalis* gingipains upregulate osteopontin (OPN), SMemb, and S100A9 expression, which are involved in the increase of VSMCs proliferation rate and enhance the capability of migrating of VSMCs through the upregulation of angiopoietins 2 (Angpt2) and ETS proto-oncogene 1 (ETS1) while inhibiting Angpt1. ETS1 is the transcription factor of Angpt2, which is essential for *P. gingivalis* to induce Angpt2 [84, 85, 86, 87]. The periodontal pathogen can produce the calcification of VSMCs and induce them to submerge lipids to form foam cells [81].

As previously mentioned, *P. gingivalis* can elucidate the immune system to manage to survive inside the host. It interacts with macrophages, T cells and dendritic cells. *P. gingivalis* enters macrophages due to the interaction with the complement receptor 3 (CR3), once *P. gingivalis* is inside, the cell activates TLR2 by its surface fimbriae and starts the signaling pathway from the inside out to cause a different conformation of CR3 with high affinity. The internalization allows *P. gingivalis* to survive and to maintain its virulence [81]. *P. gingivalis* activates the inflammatory response of macrophages; its fimbriae stimulate monocytes and macrophages to secrete pro-inflammatory cytokines, including IL-1β, IL-18, TNF-α, and NLRP3 inflammasome activation [15]. In addition, *P. gingivalis* induce macrophages to produce foam cells, which are decisive in the pathogenesis of atherosclerosis.

The periodontal pathogen creates a Th17/Treg imbalance that increases the inflammation of the atherosclerosis plaque and its instability. It also inhibits T cells through the control of differentiation and activation caused by chemokines, proliferation, and communication in T cells to guarantee their own survival, keeping them in atherosclerosis plaque and enhancing the progression of the disease. The minor fimbria of *P. gingivalis* binds with a cell adhesion molecule on Dendritic cells called CD209 to make it able to avoid immune system control, providing survival for itself and the host cell [81].

In summary, *P. gingivalis* is the periodontal pathogen that is most represented in the dental plaque of periodontal patients and is among the oral bacteria that influence atherosclerosis. It is also the most studied and represented. It can lead to arterial endothelial dysfunction, provoke foam cell generation, and make vascular smooth muscle cells proliferate and calcify.

#### 4.2.2 New Perspectives on the Molecular Interaction between Periodontal Bacteria and Endothelial Cells

In general, there are some molecular mechanisms shared by all periodontal bacteria involved in the pathogenesis of atherosclerosis. All periodontal pathogens have several pathogen-associated molecular patterns (PAMPs), including CpG DNA, lipopolysaccharide and Peptidoglycan. Such PAMPs, after being recognized by the pattern recognition receptors (PRRs) of the infected cells, start inflammatory and immune responses. The inflammatory and immune reactions cause damage to the periodontal tissues, which produces a big quantity of damage-associated molecular patterns (DAMPs), including neutrophil extracellular traps (NETs), high mobility group box 1 (HMGB1), alarmins (s100 protein). These DAMPs can enhance the evolution of atherosclerosis [88].

CpG DNA is a kind of DNA sequence which can activate the immune response through TLR9; this receptor is expressed at elevated concentrations in periodontitis-affected gingival tissue, indicating the involvement of CpG DNA in periodontal disease [89, 90, 91]. When oral pathogens manage to enter the blood stream, they can colonize the endothelium and the CpG DNA present after bacterial cell lysis may regulate the progression of Atherosclerosis by triggering the corresponding TLR9 pathway.

Lipopolysaccharide (LPS), also known as endotoxin, is a specific virulent protein of the outer membrane of negative gram bacteria, shared by all the periodontopathogens. The role of *P. gingivalis* LPS has already been discussed, but the potential role of LPS of other periodontopathogens, such as *T. denticola* and *T. forsythia *are still under investigation.

Peptidoglycan (PGN) is a component of the bacterial wall cell, including of periodontopathogens’ walls cells. There are few data about the possible correlation between periodontal PGN and atherosclerosis progression, but when periodontal pathogens colonize blood vessels, PGN can produce chronic inflammation that, if prolonged, may lead to atherosclerosis. It has been demonstrated that PGN leads to high levels of pro-inflammatory cytokines through TLR2, causing worsening of atherosclerosis. Through TLR2 and NF-kB pathways, TLR2 activates monocytes to overexpress intercellular adhesion molecule-1 (ICAM-1). In this way it enhances monocyte adhesion and chemotaxis, that lead to vascular disease PGN recognition protein-1 (PGLYRP-1) which can also recognize PGN. It should be highlighted that high levels of PGLYRP-1 are associated with vascular disease and may favor the formation of atherosclerotic plaques by modulating the overexpression of adhesion molecules [92, 93, 94, 95, 96, 97, 98, 99].

When neutrophils are stimulated, they produce an extracellular fibrous network structure called Neutrophil extracellular traps (NETs), which are constituted of chromatin and cellular proteins. The function of NETs is to block bacteria and their products, and the correlation between NETs and periodontitis has been assessed. However, the studies about the correlation between NETs and Atherosclerosis are still lacking [100, 101], buthere are some studies that support the existence of such a correlation. In fact, when *P. gingivalis* reaches the blood circulation it binds to erythrocytes avoiding ROS destruction, activating the Rho GTPase signaling pathway, up-regulating CD11b/CD18, and promoting the activation of neutrophils. *T. forsythia* is associated with the augmented relapse of NETs, intraplaque hemorrhage and neutrophil activation [102, 103].

High mobility group box 1 (HMGB1) is a non-histone chromosome binding protein that stabilizes the structure of nucleosomes, modulates the transcription factors and provides DNA replication repair. It can be released by necrotic and destructed cells and activated immune cells.

It reaches high levels in the gingival crevicular fluid during periodontal disease, stimulating macrophages in producing cytokines causing an inflammatory environment that destroys tissues.

It can enter the bloodstream and cause damage to the endothelium. There are some animal studies that suggest the correlation between high levels of HMGB1 in periodontal patients and atherosclerosis progression, but they are still insufficient, and the underlying mechanisms are still unknown [104, 105, 106, 107, 108, 109].

Alarmins are a group of calcium- binding proteins, combining PRRs with the function of activating immune cells and endothelial cells to promote inflammation. During periodontal disease, some Alarmins increase, [110] causing tissue damage. Moreover, in some animal models, the periodontal-induced increase of alarmins has been associated with atherosclerosis [111, 112, 113].

Among PRRs, Troll-like receptors (TRLs) are involved in the periodontal infection, recognizing LPS, CpG DNA etc., of the periodontopathogens and activating an aberrant inflammatory response. The chronic inflammatory signals are transferred to the bloodstream, so innate immunity in the vascular compartment is activated and contributes to the development of atherosclerosis. Macrophages have TRLs and after the recognition of LPS, CpG DNA secreted more inflammatory mediators and adhesion molecules that induce the formation of the atherosclerotic plaque [114].

Nucleotide oligomerization domain (NOD)-like receptors (NLRs) have similar action of TLRs. They are involved in periodontal infection since the periodontopathogens induces a higher expression of NOD1 and NOD2 in periodontal tissues. Once they are activated, they activate the NF-kB, and mitogen-activated protein kinase (MAPK) pathways. *P. gingivalis* up-regulates NLRs of the endothelium. In particular, NLR family pyrin domain containing 3 (NLRP3) is important in the mediation of periodontal infection in atherosclerosis, for example, it was seen that after periodontal treatment, NLRP3 was significantly reduced in gingival tissues. It reaches high serum levels in periodontal patients, and such concentrations are related to the levels of IL-1β and IL-18. The detail of the mechanisms should be further investigated [88, 115, 116, 117, 118, 119].

As the periodontal infection and inflammation progress, pathogen associated molecular patterns (PAMPs) and damage-associated molecular patterns (DAMPs), can be released into the circulation, causing the start of other inflammatory diseases, including atherosclerosis. Such molecular patterns may be targeted by inhibitors in order to turn off the inflammation and interfere with the progression of atherosclerosis. For example, Synthetic Anti-lipopolysaccharide Peptides (SALPs) can be used as inhibitors of PAMPs, like LPS, having higher affinity than LPS-binding protein, in this way the infection may be arrested. In particular, it indues inhibition on LPS-induced TNF-α secretion in monocytes. DNase may represent other molecular pattern neutralizers since PAMPs and DAMPs have acid nucleic composition and can be targeted by it. It has been seen that DNase reduce bone resorption in periodontitis, and it has, in animal models, also given good results in atherosclerosis development [88].

Since PAMPs are released during periodontal infection and DAMPs can reach the circulation inducing vascular damage, they have become therapeutic targets to stop the infection and inflammation. Some molecules have been tested in order to interrupt the relationship between molecular patterns and relative PRRs and stop the downstream transduction pathway that leads to damage to the tissues, but they are only at the beginning. This suggests that further investigation is needed to better understand the molecular mechanisms and give birth to new therapeutic agents targeting such molecular patterns [88].

The best way to know how periodontal pathogens act, inducing endothelial damage, is through *in vitro* studies. The mainly used *in vitro* models have the limitations of not representing the environmental conditions the cells normally are under. In fact, they consist of 2 dimensions (2D) cultured cells organized in a monolayer. For this reason, the physiological interaction of endothelial cells with the blood stream, with other cells floating into the blood and with extracellular elements, is not replicated. This aspect limits the correct interpretation of the molecular mechanisms induced by the oral pathogen infection and relative endothelial cell responses; they are surely different from those of cells inside the tissue. Moreover, 2D models of cells also have different cell shapes, altered proliferation rates, and altered cell phenotypes after the first passage; this augments the difficulty of having reliable considerations about their behaviour. To avoid such problems, 3 dimensions (3D) cells models have been introduced [120, 121, 122]. 3D models allow us to study cell behavior which replicates that of physiology; in fact, it is characterized by an extracellular matrix created with natural components, like collagen, that simulates the physiological environment. In 3D models, the following features of cells: adhesion, growth, proliferation, morphology, cell–extracellular matrix interactions, matrix remodeling, soluble factor availability, mechanotransduction, cell–cell interactions, cell migration, secretion of proteins and cytokines, and cell behavior and responses are more accurate and similar to the reality in the human body than in 2D models [120, 123]. In 3D models, endothelial cells adapt their behavior to the environment, considering its mechanical properties; such mechanisms couldn’t be done in 2D models [121]. In terms of the role of oral pathogens in endothelial damage, there are not enough studies in 3D models. There is a study about Infective endocarditis, a morbidity affecting cardiac valves, related to the infection of many oral bacteria. These bacteria manage to go into the blood torrent via ulcers from the oral cavity and colonize the cardiac valve. The main endocarditis related oral species are: *S. aureus*, *S. sanguis*, *S. epidermidis*, *P. gingivalis*, *Enterococcus faecalis*, *Actinobacillus actinomycetemcomitans*. This study evaluated the response of endothelial cells after infection of *S. aureus*, *S. sanguis*, *S. epidermidis* in 3D models. It demonstrated the importance of the extracellular matrix components. In fact, a reduced inflammatory response was detected in cells cultured in collagen and fibrin matrices infected by *S. aureus. *In addition, a decrease in secretion of monocyte chemotactic protein (MCP)-1 and IL-8 and monocyte adhesion in the collagen matrix was observed compared to the fibrin matrix or no extracellular matrix. A reduction of procoagulant activity of cells in collagen matrix and fibrin matrix was also reported. These results demonstrated that the endothelial cells’ response to bacterial infection are based on the extracellular matrix, which should be considered when the function of these cells is studied to have the highest accurate analysis possible [124]. The literature lacks studies in 3D models that better clarify endothelial cell behaviour after infection with periodontal pathogens. Given the importance of the extracellular matrix, future research should focus on this direction. In this way it will be easier to interfere with the pathogenesis of atherosclerosis, and maybe other therapeutical approaches would rise.

#### 4.2.3 Impact of Oral Pathogens on the Main Cardiac Conditions; Molecular Patterns and Treatment Repercussions

Hypertension is a disease affecting 30% of the adult population worldwide and is traditionally associated with the renin-angiotensin-aldosterone system (RAAS) and the sympathetic nervous system (SNS). Nevertheless, there are cases of treatment failure targeting RAAS and SNS that suggest that other factors may be implicated in the development of hypertension. It has been demonstrated that inflammatory conditions are involved in the pathogenesis of hypertension [125].

Periodontitis is a chronic inflammatory disease, and recent evidence has identified an existing correlation between periodontitis and hypertension [126] and has shown the benefit of periodontal treatment on hypertension. A decrease of systolic blood pressure (BP) has been observed related to ameliorated periodontal status and improvement of diastolic BP and endothelial function after intensive periodontal therapy in hypertension-affected patients [127]. Such data suggest the role of periodontal pathogens in the development of hypertension. In a study on animal models, it was tested whether *P. gingivalis* had effects on increasing BP. They detected that *P. gingivalis* antigen stimulation enhanced the activation of systemic T-cells, which is a characteristic of hypertension. *P. gingivalis* induced an increase of Th1 cytokines, IFN-γ and TNF-α and the T-helper-type 1 immune responses were correlated to a big elevation of BP and endothelial dysfunction [128]. Given that this was just a preliminary study; further investigations need to be done to better understand the molecular mechanisms underlying such correlation, which may eventually lead to microbiological therapies.

Also, for coronary heart disease (CHD) there have been some suggestions about the impact of periodontitis. In particular, itscorrelation with periodontal pathogens. A high concentration of *A. actinomycetemcomitans* and *P. intermedia* was detected in the subgingival plaque of patients with coronary heart disease suggesting its involvement in the progression of such diseases, besides periodontitis [129]. In patients affected by CHD and control participants, the serum levels of antigens against *P. gingivalis* and/or against *A. actinomycetemcomitans* was analysed. The study revealed higher levels of anti- *P. gingivalis* and anti- *A. actinomycetemcomitans *in patients affected by CHD, suggesting, once more, the involvement of periodontal bacteria in CHD development. It is actually unknown which molecular mechanisms driven by such periodontal pathogens drive the progression of CHD; for this reason, further studies should be completed.

Atrial fibrillation (AF) may be associated with inflammation, and since oral infections often lead to chronic inflammation, they can be associated with the development of atrial fibrillation. It was recently discovered that *P. gingivalis* secretes up to 250 proteins and *A. actinomycetemcomitans* secretes 179 different proteins, some of which may impact the remodeling of cardiac tissues and cause AF [130, 131].

Once oral bacteria go into circulation through inflamed sites of the oral cavity, they invade the heart. The sites of oral inflammation induce systemic inflammation by the relapse of inflammatory mediators into the blood stream, which affects ventricular remodeling. The host immune response to specific components of oral pathogens causes autoimmunity against molecular structures expressed in the heart, including heat shock protein 60/65 (HSP60/65) and citrullinated cardiac proteins. These are the effects related to specific bacterial proteins and toxins, such *P. gingivalis,* PAP and leukotoxin A (LtxA) that are produced by oral pathogenic bacteria and cause the formation of anticitrullinated protein antibodies (ACPA).

It is well known that poor oral hygiene is related to a high risk of developing endocarditis [132, 133].

The bacterial species which were identified in infective endocarditis-affected patients are viridans group streptococci (up to 70%) and *A. actinomycetemcomitans, *which represent the typical cause of endocarditis, but also non-typical related endocarditis bacteria, including Porphyromonas spp. (*P.s gingivalis*) (up to 50%), Actinomyces spp. (up to 30%) and Fusobacterium spp. (up to 30%). A good way to prevent such morbidity was associated with antiseptic procedures, such as rinsing with chlorhexidine, povidone-iodine or essential oils, diode laser or systemic antibiotic prescription [133].

Regarding treating such diseases, targeting the involved oral microbes, mechanical removal of the supra and subgingival plaque, systemic antibiotics, probiotics exert a promising role.

Probiotics are a promising therapeutic approach to treating oral microbiota unbalance [134].

Probiotics are gaining importance in the treatment of many diseases – in fact, it was detected that the intake of such food supplements is related to improving a lot of cardiovascular risk markers, such as LDL, cholesterol, CRP and cytokines involved in the inflammatory reaction [135]. For this reason, probiotics should be further investigated to better clarify its potential clinical application in treating cardiovascular diseases related to periodontitis.

## 5. Biomarkers and Cardiovascular Outcomes

To assess the impact of periodontal therapy in cardiovascular disease, the blood levels of the following biomarkers were evaluated at every follow-up. Nevertheless, it should be analysed if such biomarkers have sufficient strong relevance to the pathophysiology of cardiovascular disease to properly assess the efficacy of periodontal treatment.

### 5.1 Lipid Profile

Several large clinical trials have provided strong evidence supporting cholesterol’s involvement in atherosclerosis pathogenesis. The primary type of cholesterol implicated in atherosclerosis is LDL cholesterol, often referred to as “bad” cholesterol. Elevated levels of LDL cholesterol in the blood can lead to the deposition of cholesterol in the arterial walls, initiating the formation of atherosclerotic plaques.

Here are a few examples of significant clinical trials that have contributed to our understanding of cholesterol’s role in atherosclerosis: The Framingham Heart Study: This landmark study, initiated in 1948, followed a large cohort of participants over several decades. It demonstrated a strong association between high blood cholesterol levels, particularly LDL cholesterol, and the development of atherosclerosis and subsequent cardiovascular events [136].

The Scandinavian Simvastatin Survival Study (4S): This trial, conducted in the 1990s, involved patients with preexisting coronary heart disease. It showed that statin therapy, which lowers LDL cholesterol levels, significantly reduced the risk of cardiovascular events and mortality, providing direct evidence that reducing cholesterol can improve outcomes in individuals with atherosclerosis [137, 138].

The Heart Protection Study (HPS): This trial enrolled a large number of high-risk patients, including individuals with preexisting vascular disease or diabetes. It demonstrated that treatment with a statin (simvastatin) significantly reduced major cardiovascular events, again highlighting the role of cholesterol management in preventing atherosclerosis-related complications [138].

The Cholesterol Treatment Trialists’ (CTT) Collaboration has conducted several meta-analyses of randomized controlled trials evaluating cholesterol-lowering interventions. Their analyses consistently showed that lowering LDL cholesterol levels with statin therapy substantially reduces the risk of major cardiovascular events across various patient populations [138].

These and other clinical trials have provided robust evidence supporting the involvement of cholesterol, particularly LDL cholesterol, in the development and progression of atherosclerosis. They have played a crucial role in shaping clinical guidelines for managing cholesterol levels and reducing cardiovascular risk. For this reason, there are no doubts about the validity of the usage of lipid profiles as biomarkers to evaluate cardiovascular outcomes. If periodontitis treatment manages low blood levels of such lipid biomarkers, we can confidently conclude that periodontal treatment positively influences cardiovascular health.

### 5.2 C-Reactive Protein

C-reactive protein (CRP) is a biomarker of disease activity that is widely used in various medical conditions. Although it is primarily known as an inflammatory marker, CRP also has functions that can directly impact the inflammatory response in the body. The main role of CRP is an inflammation marker: it is produced by the liver in response to inflammation. Its levels increase rapidly during acute inflammatory conditions, such as infections, tissue injury, or autoimmune disorders. Measuring CRP levels can provide valuable information about the presence and intensity of inflammation in the body [139, 140]. It is involved in opsonization and phagocytosis: CRP plays a role in the immune response by binding to certain microbial pathogens, damaged cells, or foreign particles. This process, called opsonization, marks these targets for recognition and engulfment by phagocytic cells, such as macrophages. By enhancing phagocytosis, CRP helps to eliminate pathogens and damaged cells, contributing to the resolution of inflammation. CRP can also activate the complement system, which is part of the innate immune response [141]. Activation of the complement system triggers a cascade of reactions that enhance inflammation, attract immune cells to the site of injury or infection, and help in the elimination of pathogens. This activity of CRP links it to the broader immune response beyond its role as an inflammation marker. CRP can impact endothelial function and contribute to the development of atherosclerosis and cardiovascular diseases. CRP can interact with endothelial cells that line blood vessels. It has been shown to influence the expression of adhesion molecules and chemokines on endothelial cells, leading to the recruitment of immune cells to the site of inflammation [139].

While CRP is primarily used as an indicator of inflammation, it also directly affects immune responses and endothelial function. These functions suggest that CRP may be more active in shaping the inflammatory process and its associated pathologies. However, it is important to note that the precise mechanisms and implications of CRP’s functions in various diseases are still an active area of research. Due to its multiple functions, CRP may be considered to have low specificity for cardiovascular risk assessment. Many studies have analysed its role in predicting cardiovascular risk and atherosclerosis progression. Among those studies, there was a focus on assessing the strength of CRP evaluation in cardiovascular monitoring, but there were also controversial studies that showed the weakness of CRP [142, 143]. The different study designs or small samples may have caused the data discrepancy. Over the years, the role of CRP in cardiovascular monitoring has gained more validity and strength. For example, the study of Ridker *et al*. [144] demonstrated the power of CRP in predicting future cardiovascular events. Moreover, compared with lipid profile, CRP showed higher relevance, suggesting that lipid profile alone may be considered weak cardiovascular risk predictor but can be stronger if associated with CRP.

### 5.3 Interleukin-6

Interleukin-6 (IL-6) is a pro-inflammatory cytokine with pleiotropic function. IL-6 acts in a wide range of homeostatic processes, influencing various physiological and pathological conditions. It is involved in lipid metabolism, insulin resistance, mitochondrial activities, the neuroendocrine system, neuropsychological behaviour, and systemic conditions. IL-6 plays a role in lipid metabolism by regulating the production and breakdown of lipids. It can stimulate the release of fatty acids from adipose tissue, influence lipid oxidation, and impact cholesterol metabolism. The dysregulation of IL-6 in lipid metabolism has been implicated in conditions such as obesity and dyslipidemia. Another function of IL-6 is that it can contribute to the development of insulin resistance, a key factor in the pathogenesis of type 2 diabetes. Elevated levels of IL-6 have been associated with impaired insulin signaling and glucose uptake in tissues, potentially leading to insulin resistance and glucose intolerance. IL-6 has also been shown to influence mitochondrial function and biogenesis. It can affect energy metabolism by regulating oxidative phosphorylation and mitochondrial respiration. Dysregulation of IL-6-mediated mitochondrial activities may contribute to metabolic disorders and age-related diseases. Moreover, it is involved in communication between the immune and central nervous systems. It can modulate the activity of the hypothalamic-pituitary-adrenal (HPA) axis, which plays a role in stress response and neuroendocrine regulation. IL-6 has also been implicated in mood disorders, cognitive impairment, and neurodegenerative diseases. IL-6 is associated with various systemic conditions, including cardiovascular disease and rheumatoid arthritis. In cardiovascular disease, IL-6 is involved in the inflammatory processes that contribute to atherosclerosis and plaque instability. In rheumatoid arthritis, IL-6 plays a central role in the inflammatory cascade, contributing to joint inflammation and destruction [145, 146, 147, 148].

The pleiotropic actions of IL-6 highlight its involvement in numerous physiological processes and its impact on various diseases. Understanding these diverse functions of IL-6 is important for developing targeted therapeutic interventions and managing conditions where IL-6 dysregulation is implicated.

In physiological conditions, the serum concentration of IL-6 is 1–5 pg/mL, which can greatly increase in pathological conditions [149]. Interestingly, in individuals who have a genetic variation, meaning the concentration of IL-6- receptors is high but IL-6 cell signaling is low, the coronary heart disease risk is decreased [150, 151]. Assessed by its role in cardiovascular disease, IL-6 can be a good biomarker in determining cardiovascular disease risk.

### 5.4 Surrogate Vascular Outcomes

The previous biomarkers have been recognized to be involved in cardiovascular risk, but there is a limitation. In fact, such biomarkers are non-specific for vascular inflammation, and they may also increase in other pathological conditions, causing bias in the interpretation of cardiovascular disease. It is certain that they still are valuable tools to detect cardiovascular health, but to better estimate the conditions of the cardiovascular system the evaluation of such biomarkers must be completed with the employment of non-invasive imaging.

The assessment of intima-media thickness (IMT) of the carotid artery using B-mode ultrasound has emerged as a valuable tool for evaluating the presence and progression of atherosclerosis and estimating future cardiovascular risk. This non-invasive imaging technique has gained popularity and is widely used in both clinical and research settings. Here are some key points regarding the use of carotid IMT measurement:

(1) Atherosclerosis assessment: Carotid IMT measurement helps evaluate the thickness of the innermost two layers of the carotid artery wall: the intima (innermost layer) and the media (middle layer). An increased IMT is associated with early-stage atherosclerosis and can indicate the presence of subclinical vascular disease [152].

(2) Cardiovascular risk estimation: Carotid IMT measurement provides information about an individual’s cardiovascular risk. Studies have demonstrated that greater IMT values are associated with an increased risk of future cardiovascular events, such as heart attacks and strokes. By identifying individuals with subclinical atherosclerosis, carotid IMT assessment helps identify those at higher risk who may benefit from aggressive preventive measures. The Bogalusa Heart Study demonstrated the correlation between carotid intima-media thickness and cardiovascular disease from young age [153].

(3) Noninvasive and safe: B-mode ultrasound is a noninvasive imaging technique that uses high-frequency sound waves to create carotid artery images. It is a safe and well-tolerated procedure suitable for repeated assessments and follow-up examinations. It does not involve radiation exposure or the need for contrast agents, making it a preferred choice for evaluating atherosclerosis in clinical practice [152, 154].

(4) Clinical and research applications: Carotid IMT assessment is used in various clinical settings. It is commonly performed as part of cardiovascular risk assessment in individuals with risk factors for atherosclerosis, such as hypertension, diabetes, or dyslipidemia. Carotid IMT measurement can also be used to monitor disease progression or the effectiveness of interventions aimed at reducing atherosclerosis [152, 155].

Additionally, carotid IMT measurement has been employed in numerous research studies investigating the relationship between atherosclerosis and various factors, such as genetics, lifestyle, and therapeutic interventions. It has contributed to our understanding of the pathophysiology of atherosclerosis and the development of novel preventive and treatment strategies [156].

In summary, the assessment of carotid IMT using B-mode ultrasound is a non-invasive, safe, and well-tolerated technique that has proven invaluable in evaluating the presence and progression of atherosclerosis and estimating future cardiovascular risk. Its adoption in both clinical and research settings has facilitated risk stratification, early detection of subclinical disease, and monitoring of therapeutic interventions.

In addition, another aspect that has been suggested to have predictable value in cardiovascular risk is represented by the evaluation of endothelial function. Inaba *et al*. [157] have shown the correlation between the measurement of endothelial function and future cardiovascular events by a non-invasive exam, that is, flow-mediated dilatation (FMD) of the brachial artery. The results showed that the damage of brachial FMD is significantly associated with future cardiovascular events. It was also demonstrated by Matsuzawa *et al*. [158] that non-invasive peripheral endothelial function tests, including FMD and reactive hyperemia peripheral arterial tonometry (RH-PAT), manage to successfully predict future cardiovascular events. In summary, surrogate cardiovascular outcomes are a valuable tool in predicting and monitoring cardiovascular events, giving a more accurate evaluation than lipid and inflammatory biomarkers alone.

## 6. Impact of Periodontal Treatment on Cardiovascular Disease

Periodontal therapy consists of the intervention of its causing factors. Obviously, it is impossible to interfere with genetic predisposition and familiarity, but it is possible to interfere with the other causing factors. As assessed by the most recent guidelines by Sanz *et al*. [159, 160] the periodontal treatment is based on the identification of the stage, in fact, for stages I, II and III there are common ways of intervention; on the contrary, for stage IV, beside the intervention modalities shared with the treatment of the first three stages, there is one more step of the therapy. All stages share the following therapeutic interventions: control of the biofilm by rigorous home dental care and professional supra- and sub-gingival debridement; control of the other associated behavioral factors, for example reducing or eliminating the smoke of cigarettes and to control the other systemic diseases if present. Last but not least, an important part of the therapy is the education and the motivation of the patient at every visit to guarantee the adherence of the patient to the therapy, in fact since periodontitis is a chronic pathology, it requires a lifetime therapy, in which the clinician should often see the patient. All the aforementioned intervention approaches are valid for periodontitis patients at stage IV, but these patients also have to undergo surgical correction of bone defects. In association with the surgery, considering the severity of the stage, such patients’ condition often requires multidisciplinary interventions, such as provisional control of secondary occlusal trauma, orthodontic treatment, rehabilitation of the edentulous areas, and tooth-supported or implant-supported dental prostheses.

Since epidemiological data have assessed the correlation between CVDs and periodontitis, it is worth analysing if periodontal treatment may influence cardiovascular health, particularly the role of periodontal treatment in primary and secondary prevention of CVDs (Table 1, Ref. [161, 162, 163, 164, 165, 166]). It has been demonstrated that attention to oral health induces a decrease in the incidence of CVDs. For example, De Oliveira *et al*. [161] reported that self-performed oral hygiene habits are associated with less cardiovascular risks, in fact the subjects who brushed less than once a day had the most elevated incidence of ACVD events (high ratio (HR) = 1.7, 95% CI [1.3; 2.3]) compared with those who brushed twice a day, suggesting that home dental hygiene routines influence the incidence of ACVD. Also Park *et al*. [162] demonstrated the impact of domiciliary oral care in CVDs. They conducted a prospective population-based study handling data provided by the National Health Insurance System-National Health Screening Cohort, in which 247,696 people free from any CVD history were included. The results showed that a higher risk of future major cardiovascular events was associated with poor oral health, including an augmented number of dental caries lesions, the presence of periodontal disease and a higher rate of teeth loss; just one more episode of toothbrushing per day induced a decrease of the incidence of ACVD events (HR = 0.91, 95% CI [0.89, 0.93]) and the risk was even more reduced by regular professional instrumentation (HR = 0.86, 95% CI [0.82; 0.90]). Lee *et al*. [163] investigated the correlation between periodontal disease and acute myocardial infarction and evaluated the outcome of oral prophylaxis on the incidence rate (IR) of acute myocardial infarction; the results showed that periodontal disease is related to a greater risk of acute myocardial infarction, which can be decreased by oral prophylaxis to keep a healthy environment in periodontal supporting tissues. Sen *et al*. [164] concluded that increased self-reported dental visits reduce the risk of CVDs. Moreover, Holmlund *et al*. [165] investigated the role of professional periodontal treatment, it was observed that patients who didn’t respond well to the therapy had a greater incidence of CVDs (n = 870) when compared with responders (23.6 vs. 15.3%, *p* < 0.001), suggesting that successful periodontal treatment might influence the advancement of subclinical CVDs.

**Table 1.** **Analysis about the correlation between periodontal treatment and cardiovascular health**.

Study	Topic	Results
de Oliveira *et al*. [161]	Primary prevention	Brushing less than once a day showed the greatest incidence of ACVD events (HR = 1.7, 95% CI [1.3; 2.3])
Park *et al*. [162]	Primary prevention	Higher risk of future major cardiovascular events was associated with poor oral health
Lee *et al*. [163]	Primary prevention	Periodontal disease is combined with a higher risk of acute myocardial infarction
Sen *et al*. [164]	Primary prevention	Increased self-reported dental visits decrease the risk of CVDs
Holmlund *et al*. [165]	Primary prevention	Patients who didn’t respond well to the therapy had an augmented incidence of CVDs (n = 870) when compared with responders (23.6 vs. 15.3%, *p * < 0.001)
Offenbacher *et al*. [166]	Secondary prevention	Showed no statistically significant variation in the rate of CVD events between patients treated for periodontitis compared to community care (risk ratio = 0.72, 95% CI [0.23; 2.22])

ACVD, atherosclerotic cardiovascular disease; HR, high ratio; CVDs, cardiovascular diseases.

### 6.1 Secondary Prevention

There is still insufficient information regarding the impact of periodontal treatment on secondary prevention of CVDs. A pilot multicentre study [166, 167] showed no statistically significant variation in the rate of CVD events between patients treated for periodontitis compared to community care (risk ratio = 0.72, 95% CI [0.23; 2.22]). The results have limited real world applicability for clinicians due to several limitations of the study that need to be avoided to better clarify if periodontal treatment influences the secondary prevention of CVDs or not. Such limitations included the recruitment of a sufficient number of patients and their presence during follow-ups, which was inconsistent; moreover, it was highlighted that other factors, including obesity, may influence the outcome of the study.

Moreover, intervention trials have demonstrated that the treatment of periodontitis can lower blood inflammatory factors, enhance the lipid profile, and cause favourable modifications in various surrogate markers for cardiovascular disease. Kolte *et al*. [168] assessed the correlation between non-surgical periodontal therapy (NSPT) on serum lipid profile and cytokines in patients with stage III periodontitis. They recruited 60 patients who underwent NSPT at baseline, at 3 and 6 months. Biochemical parameters like serum lipid parameters of total cholesterol (TC), triglycerides (TG), LDL, high-density lipoprotein (HDL), IL-6 and IL-8 serum levels were assessed at baseline and 6 months post-NSPT. Periodontal probing depth (PPD) (2.75 ± 0.41), CAL (3.23 ± 0.56), lipid profile, and serum cytokine levels 6 months post-NSPT were significantly reduced (*p* < 0.0001) compared to baseline. Among the biochemical parameters, after 6 months of NSPT, a significant decrease was observed (*p* < 0.0001) of the following parameters: IL-6 (35.3%), IL-8 (41.6%), TC (7.5%), TG (1.78%), LDL (6.2%), and HDL (–21.8%). The aforementioned results indicated that since high levels of such biomarkers are associated with a high risk of developing CVD, due to NSPT in periodontitis stage III patients, which produces a decrease in them, it is possible to reduce the possibility of developing CVDs. Tawfig *et al*. [169] evaluated the changes in lipid profile and CRP levels in 30 periodontitis patients affected by hyperlipidemia. After three months of NSPT, a decrease in LDL and CRP was detected alongside good periodontal clinical outcomes. A randomized trial conducted by Fu *et al*. [170] involved 109 periodontitis patients affected by hyperlipidemia reported inferior levels of triglycerides and circulating pro-inflammatory cytokines and greater levels of HDL in the group of patients treated with intensive periodontal therapy. Hada *et al*. [171] investigated the effects of NSPT on cardiovascular disease markers in a randomized trial involving 55 patients; the results assessed a significant decrease of very low density lipoprotein (VLDL) in patients treated with NSPT. Since lipid profile has been demonstrated to have an important impact on cardiovascular risk, in particular, 1 mmol/L decrease in the level of low-density lipoprotein cholesterol is related to a 12% reduction in all-cause mortality and a 19% decrease in cardiovascular mortality [172], the aforementioned studies suggest the presence of correlation between periodontal therapy and lipid levels and, in the end, with cardiovascular health; nevertheless, further studies need to be done to assess the existence of such correlation in bigger numbers of participants.

CRP seems to be a good biomarker to assess cardiovascular risk [173] and periodontitis may induce a rise of CRP levels, inducing a systemic inflammatory condition that, in the end, enhances the cardiovascular risk. In the literature, there are controversial results about the impact of the therapy of periodontitis on CRP concentrations and insufficient data due to the deficit of appropriate long-term, well-designed, randomized controlled trials [156]. In fact, Souza *et al*. [174] did a non-randomized trial and demonstrated a significant decrease of CRP concentration after 60 days of periodontal therapy in patients who had more than 3 mg/L at baseline; Gupta *et al*. [175] demonstrated a reduction of the levels of CRP in chronic and aggressive periodontal patients from 3.03 ± 1.67 to 1.46 ± 1.67 mg/L and from 3.09 ± 1.21 to 1.43 ± 1.21 mg/L, respectively, 3 months after periodontal treatment. On the contrary, Almaghlouth *et al*. [176] and Eickholz *et al*. [177] did not find any reduction of CRP levels in periodontal patients who underwent periodontal therapy 3 months before. Nevertheless, based on the available evidence, it can be assessed that periodontal therapy induces a reduction of CRP levels, associating it with a lower cardiovascular risk [156].

IL-6 is another inflammatory mediator which has been found to reach high levels in periodontitis and cardiovascular disease. Some studies showed differences between periodontal subjects and healthy controls, in fact in the first group higher IL-6 concentrations were detected, which were reduced following periodontal therapy [156].

Periodontal therapy has been shown to influence surrogate vascular imaging outcomes, including carotid intima-media thickness, which assesses the existence and advancement of atherosclerosis and evaluates future risk of cardiovascular events and endothelial function. Desvarieux *et al*. [178] reported that greater concentrations of periodontopathogens were cross-sectionally correlated with thicker carotid intima-media thickness. The same group of scientists documented parallel changes in periodontal and longitudinal health carotid artery intima-media thickness progression during 3 years [179]. Kapellas *et al*. [180] in a randomized controlled trial reported a decrease in carotid intima-media thickness after 12 months of periodontal therapy in periodontal patients. The results showed a statistically significant variation in intima-media thickness change between treatment groups (–0.026 mm, 95% CI –0.048 to –0.003 mm; *p* = 0.03).

Regarding endothelial function, many intervention trials demonstrated the benefit of periodontal therapy. For example, in the study of Tonetti *et al*. [181] it was shown that the test group had a 2% greater value of fibromuscular dysplasia (95% confidence interval 1.2–2.8%; *p* < 0.001) compared with controls after 6 months of intensive periodontal therapy.

### 6.2 Impact of Periodontal Treatment Approaches: Limitations and Potential for Improving Patient Outcomes

According to the aforementioned studies, the domiciliary oral care and the professional instrumentation consistent with frequent dentist visits to maintain oral health are essential to maintain cardiovascular health since they have been associated with lower incidence of cardiovascular events. For periodontal patients, the success of periodontal therapy is the key. In fact, as demonstrated by Holmlund *et al*. [165], unsuccessful periodontal treatment was associated with the highest incidence rate of CVDs. It is necessary to clarify what makes periodontal therapy successful or not. In the study of Holmlund *et al*. [165] the adopted therapy was nonsurgical therapy by ultrasound or manual instruments, 1 quadrant at a time. After 6–8 weeks, they reevaluated the patients and if necessary, a nonsurgical treatment was performed once again for the residual pockets, or surgical correction of bone defects was done. Among the factors that may be responsible for the failure of therapy in some patients, it was suggested that smoke didn’t correlate with the response to treatment (*p* = 0.80). The factor that influenced the success of the therapy was the number of periodontitis-affected teeth; in fact, there was an increase of the incidence of cardiovascular risk, from 1.28 (95% CI, 1.07 to 1.53) to 1.39 (95% CI, 1.13 to 1.73), between those having >0 teeth and those with >20 teeth. Thus, one limitation of periodontal treatment may be related to the degree of severity of the patient’s initial condition.

All the studies that showed good cardiovascular clinical outcomes suggested the effective correlation between periodontal therapy and cardiovascular health. The periodontal treatment approaches performed were supragingival and subgingival plaque and mechanical calculus removal. Some studies compared supragingival instrumentation with subgingival instrumentation, also referred to as intensive periodontal therapy. The second one was more efficient since it included a more accurate removal of the etiological factor of periodontitis. Thus, it is translated into more control of the infection and inflammation of periodontal tissues, which has a good influence on the blood levels of inflammatory biomarkers. Intensive periodontal therapy may sometimes be associated with adjunctive therapy with systemic or local drugs. The most used drugs as an adjunct to periodontal therapy are antibiotics. Firstly, they are administrated systemically, and they have also been associated with lower CRP levels in periodontitis patients [177]. Nevertheless, antibiotic systemic administration was associated with some controversial effects, including gastrointestinal issues, bacterial resistance and the need for frequent doses to reach a sufficient concentration in the periodontal pockets since they are metabolized by the liver or kidney [182]. Local administration of drugs allows avoiding such problems. Locally administrated antibiotics have shown good periodontal outcomes, but they can’t be used for long term due to their side effects [183]. For this reason, new therapeutical agents, such as host modulators and natural agents, are arising and their efficacy is still under investigation [182]. If their effectiveness in periodontal treatment was assessed, it would be interesting to test their influence on cardiovascular outcomes.

## 7. Conclusions and Future Perspectives

Periodontal disease and cardiovascular health are closely related. Considering the reported evidence in our review, periodontal therapy has a good influence on blood inflammatory mediators, it enhances the lipid profile, and causes favorable changes in various surrogate markers for cardiovascular disease. Such reported data suggest that periodontal treatment may minimize the risk for cardiovascular disease, even though we suggest that new intervention trials should be conducted to better clarify the exact underlying mechanisms that enhance cardiovascular health after periodontal therapy. Moreover, considering the existing correlation between Periodontal disease and CVDs, dentists and cardiologists should collaborate to favour primary prevention and provide personalized therapy for cardiopathic patients, periodontal patients and cardiopathic and periodontal patients; in this way the incidence and the related complications will be reduced.
